# Synthesis and Design of a Miniaturized Broadband Bandstop Filter with a Simple Structure

**DOI:** 10.3390/mi16060607

**Published:** 2025-05-23

**Authors:** Chuan Shao, Rong Cai, Xinnai Zhang, Kai Xu

**Affiliations:** 1 School of Information Engineering, Jiangsu College of Engineering and Technology, Nantong 226000, China; 2 Nantong Key Laboratory of Artificial Intelligence New Quality Technology, Jiangsu College of Engineering and Technology, Nantong 226000, China; 3 Research Center for Intelligent Information Technology, Nantong University, Nantong 226019, China; xukaihopeness@hotmail.com

**Keywords:** broadband bandstop filter, parallel-coupled microstrip lines, rejection bandwidth, closed-form equations

## Abstract

In this paper, a miniaturized broadband bandstop filter with a simple structure is proposed, synthesized, and developed. The proposed broadband bandstop filter is designed using asymmetrically loaded parallel-coupled microstrip lines, resulting in five transmission zeros within the stopband. Closed-form formulas of the entire set of generated transmission zeros are derived to guide a practical design procedure. To demonstrate the effectiveness of the proposed concept and synthesis method, a miniaturized broadband bandstop filter centered at 3 GHz with a 20 dB rejection bandwidth of about 100% is designed, fabricated, and measured. The core circuit size of the developed broadband bandstop filter is only 0.5 *λ*_g_ × 0.1 *λ*_g_ (31.2 mm × 6.5 mm).

## 1. Introduction

In modern microwave communication systems, the role of broadband bandstop filters has become increasingly significant. As communication systems grow more complex, interference from unwanted frequfugureency bands poses a critical challenge to signal integrity and system performance. Unlike bandpass filters [1], which allow a specific frequency range to pass through, bandstop filters are widely employed to suppress particular frequency ranges while permitting other signals to pass unimpeded. Currently, several common design methods for bandstop filters are widely used in the field of microwave communication. The most common method employed for designing microstrip bandstop filters is based on the open-circuit stub-loaded transmission line structure [2,3,4,5]. This design is straightforward and offers design flexibility. Moreover, its variants are widely utilized in harmonic suppression for bandpass filters and in RF chokes among active circuits [6,7]. However, broadband bandstop filters designed using this method often suffer from a large size, which consequently limits their application in miniaturized systems. Another frequently used method for designing bandstop filters involves the use of transmission lines with shunt-loaded resonators, which is also noted for its design simplicity [8,9,10]. This technique is also suitable for dual-band, multi-band bandstop filters and ultra-wideband bandpass filters with notched bands [11,12,13]. Nevertheless, similar to the previously mentioned approach, broadband bandstop filters designed in this way are typically bulky. In order to minimize circuit dimensions, researchers have designed a series of broadband bandstop filters based on signal interference techniques [14,15,16]. Similarly, to minimize circuit dimensions, parallel-coupled microstrip lines are commonly employed as the basis for designing broadband bandstop filters [17,18,19,20]. Furthermore, in order to continuously enhance the performance of bandstop filters, these two aforementioned methods have been innovatively integrated [21,22]. In this scenario, however, the sizes of these bandstop filters remain relatively large.

In this paper, a comprehensive design method for a compact broadband bandstop filter is proposed. The developed bandstop filter is constructed using asymmetrically loaded parallel-coupled microstrip lines, and five transmission zeros within the stopband are achieved accordingly. In order to guide a practical design, the equivalent circuit model is developed, and the closed-form equations for these transmission zeros are derived. The simulation and experimental results are in good agreement, which validates the correctness of the design theory.

## 2. Broadband Bandstop Filters

**Figure 1:**
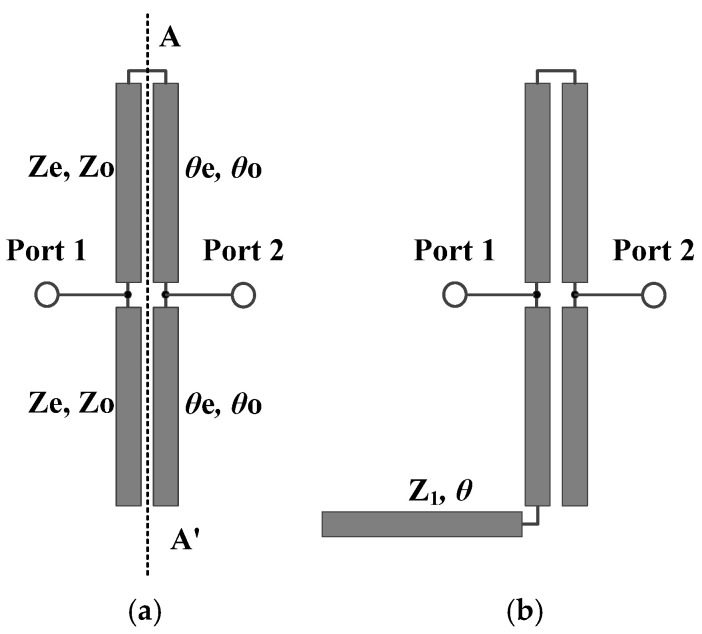
Schematics of broadband bandstop filters, (**a**) bandstop filter I, (**b**) bandstop filter II.

Schematics of the presented broadband bandstop filters are given in Figure 1. According to this figure, bandstop filter I consists of two sets of parallel-coupled microstrip lines, whereas the open-circuited sections of one set are interconnected. Bandstop filter II is derived from bandstop filter I by asymmetrically loading an open-circuited stub with quarter-wavelength length at the operating frequency. The specific parameters of these parallel-coupled microstrip lines are provided in Figure 1b. Owing to the quasi-TEM propagation mode of microstrip lines, the phase velocities of the even and odd modes in coupled microstrip lines are slightly different. This discrepancy results in slight differences between the values of *θ*_e_ and *θ*_o_. According to Reference [23], when using a Rogers 4003C substrate (a loss tangent of 0.0027, a dielectric constant of 3.38, and a thickness of 0.813 mm), the specific values of *θ*_e_ and *θ*_o_ for the quarter-wavelength coupled microstrip line are about 90.6° and 89.4°, respectively, at the center frequency. Given that the values of *θ*_e_ and *θ*_o_ are extremely close, the subsequent analysis is simplified by assuming *θ*_e_ = *θ*_o_ = *θ*.

By conducting an odd–even mode analysis on bandstop filter I, the corresponding odd–even mode input admittances can be obtained as follows:

(1) $$Y_{\mathrm{ino}}=jY_{o}(\tan\theta -\cot\theta )$$

(2) $$Y_{\mathrm{ine}}=2jY_{e}\tan\theta$$

Given the relationship between *S*_21_ and the odd-mode and even-mode input admittances (*Y*_ine_ and *Y*_ino_), we obtain the following:

(3) $$S_{21}=\frac{Y_{0}(Y_{\mathrm{ino}}-Y_{\mathrm{ine}})}{\left(Y_{0}+Y_{\mathrm{ine}})(Y_{0}+Y_{\mathrm{ino}}\right)}$$

setting *S*_21_ = 0 allows the identification of two transmission zeros (*θ*_z1_, *θ*_z2_) for bandstop filter I, which can be derived as follows:

(4) $$\theta_{Z1}=acr\tan\sqrt{\frac{Y_{o}}{Y_{o}-2Y_{e}}}=acr\tan\sqrt{\frac{1}{1-2Z_{o}/Z_{e}}}$$

(5) $$\theta_{Z2}=\pi -\theta_{Z1}$$

**Figure 2:**
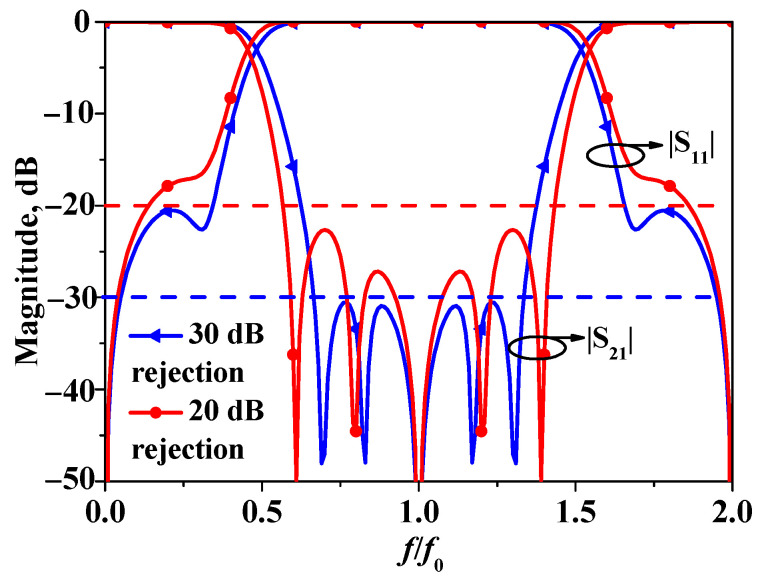
Simulated results of a bandstop filter with in-band rejection over 20 dB (Z_e_ = 90 Ω, Z_e_ = 40 Ω, Z_1_ = 120 Ω) and over 30 dB (Z_e_ = 89 Ω, Z_e_ = 41 Ω, Z_1_ = 240 Ω).

In the context of general bandstop filters, a higher in-band rejection level is typically deemed more desirable. However, it is generally challenging to achieve a high in-band rejection level while simultaneously obtaining a wide stop-band width. Figure 2 presents two design schemes. As illustrated, when the in-band rejection level is set at 30 dB, the operational bandwidth of the bandstop filter is limited to only 78%. Conversely, when the in-band rejection level is reduced to 20 dB, the operational bandwidth of the bandstop filter can reach 100%. Given that the objective of this design is to develop a wideband bandstop filter, a comprehensive evaluation of various factors has been conducted. Consequently, the one with in-band rejection over 20 dB has been selected as the optimal choice.

**Figure 3:**
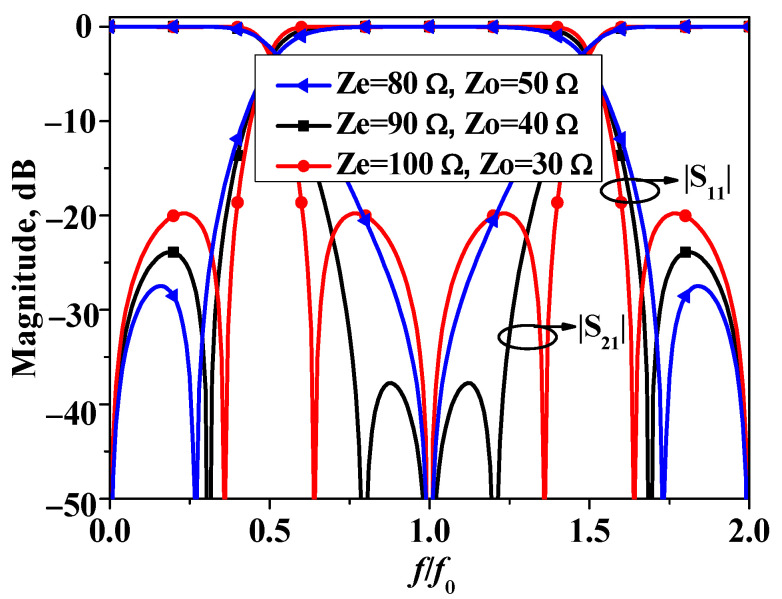
Simulated S11 and S21 of bandstop filter I versus different combination of Z_e_ and Z_o_.

Moreover, two stubs in bandstop filter I are equivalent to quarter-wavelength open-circuited stubs for the input and output ports, thereby generating an additional transmission zero at the center frequency, leading to three transmission zeros across the stopband. According to Equations (4) and (5), it can be observed that, for bandstop filter I, the positions of transmission zeros *θ*_z1_ and *θ*_z2_ are primarily determined by the odd–even mode impedance ratio. To illustrate the impact of the odd/even-mode impedances on bandstop filter I more clearly, Figure 3 presents the *S*-parameters of bandstop filter I under different combinations of odd–even mode impedances. As shown in Figure 3, the stopband attenuation level of bandstop filter I is truly determined by the ratio of the odd/even-mode impedances. Furthermore, based on the results in Figure 3 and taking into account the stopband rejection of the filter and practical fabrication tolerances, Z_e_ = 90 Ω and Z_o_ = 40 Ω are selected for the subsequent filter design.

**Figure 4:**
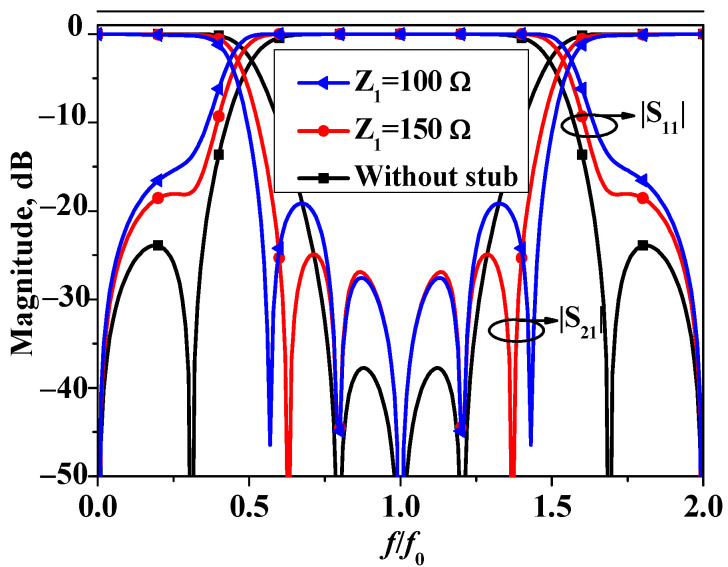
Simulated S11 and S21 of bandstop filter I and II. (Z_e_ = 90 Ω, Z_o_ = 40 Ω).

To further enhance the performance of bandstop filter I, a quarter-wavelength-length open-circuited stub with an impedance of Z_1_ is asymmetrically loaded to one of the open ends of bandstop filter I, which is shown in Figure 1b. Figure 4 exhibits *S*-parameters of the two bandstop filters under different Z_1_ values. It can be observed from Figure 4 that the introduction of the open-circuited stub results in two additional transmission zeros. This enhancement increases the stopband bandwidth of the bandstop filter without affecting the previous three transmission zeros.

**Figure 5:**
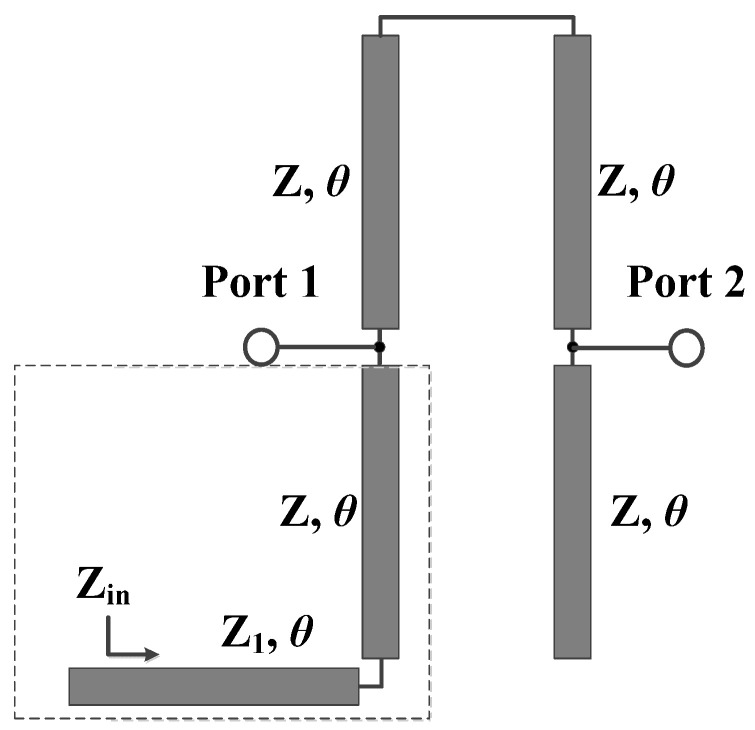
Transmission line equivalent circuit of bandstop filter II.

In order to determine the closed-form formulas for the additional transmission zeros, the transmission line equivalent circuit for bandstop filter II, based on conventional coupled microstrip line theory [24], is provided in Figure 5. According to Figure 5, the input impedance of the structure in the dashed-line box can be derived as follows:

(6) $$Z_{\mathrm{in}}=jZ_{1}\frac{Z_{1}\tan\theta +Z\tan\theta }{Z_{1}-Z\tan^{2}\theta }$$

where

(7) $$Z=\sqrt{Z_{e}Z_{o}}$$

By setting *Y*_in_ = 1/Z_in_ = 0, the following relationship can be derived:

(8) $$\theta =\arctan\left(\sqrt{Z_{1}/Z}\right)$$

Therefore, the two newly generated transmission zeros are as follows:

(9) $$\theta_{Z3}=\arctan\left(\sqrt{Z_{1}/Z}\right)$$

(10) $$\theta_{Z4}=\pi -\arctan\left(\sqrt{Z_{1}/Z}\right)$$

**Figure 6:**
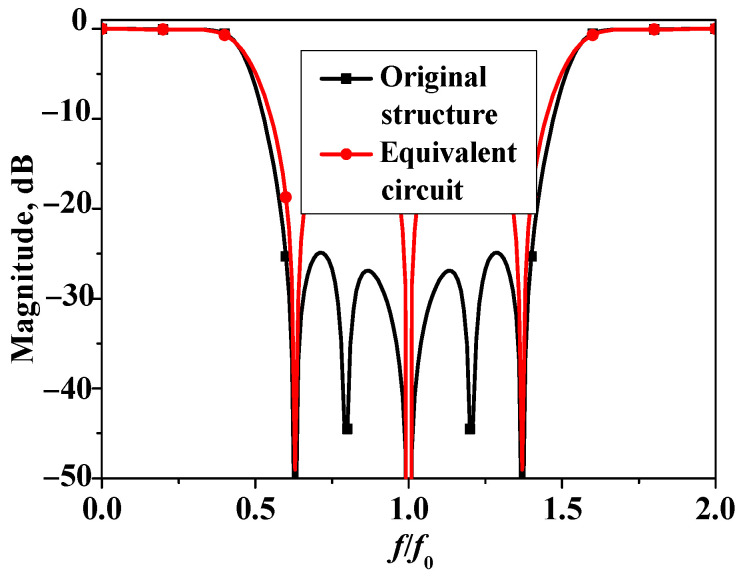
Transmission coefficients of original structure and transmission line equivalent circuit of bandstop filter II (Z_e_ = 90 Ω, Z_o_ = 40 Ω, Z_1_ = 150 Ω).

To verify the theoretical analysis and the derived formulas, Figure 6 presents the transmission coefficients of the original structure and the transmission line equivalent circuit of bandstop filter II. As shown in Figure 6, for the equivalent circuit, when the coupling between the two sets of microstrip lines is neglected, transmission zeros *θ*_z1_ and *θ*_z2_ will disappear. The positions of the three zeros in the equivalent circuit are basically consistent with those of the transmission zeros generated by the open-circuited stubs in the original structure. This validates the theoretical analysis and the derived closed-form formulas, providing a solid foundation for the subsequent fabrication and testing of the bandstop filter.

## 3. Results and Discussion

**Figure 7:**
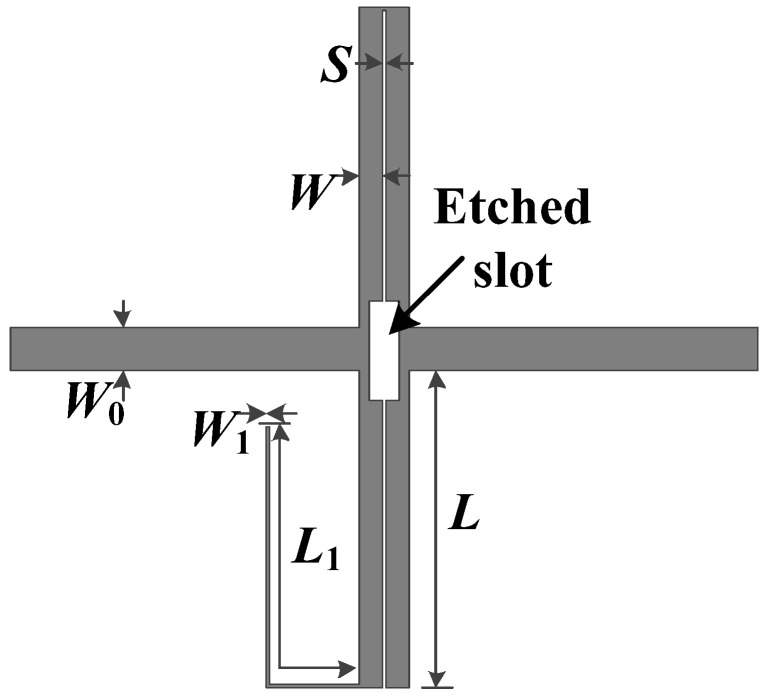
Layout of proposed broadband bandstop filter II. (*W*_0_ = 1.95 mm, *L* = 16.5 mm, *W* = 1.05 mm, *S* = 0.1 mm, *L*_1_ = 15.9 mm, and *W*_1_ = 0.2 mm).

**Figure 8:**
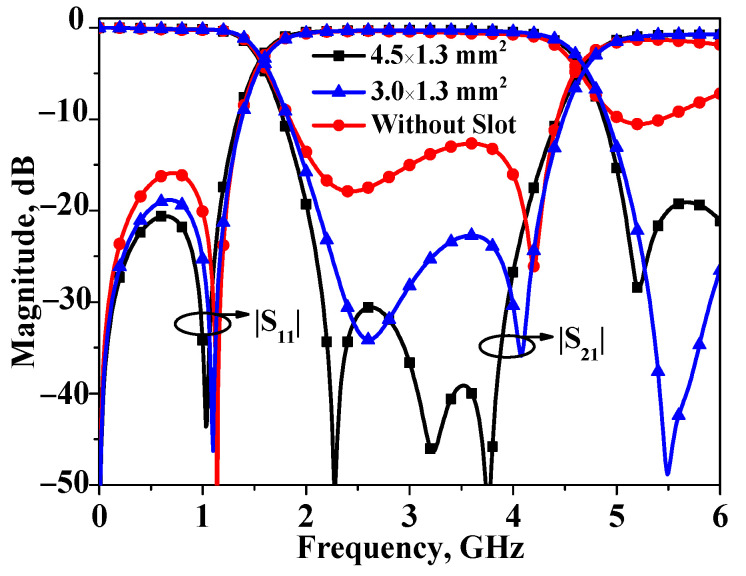
Simulated results of bandstop filter I with different sizes for the etched slot.

The layout of the proposed broadband bandstop filter II is exhibited in Figure 7. In the preceding analysis, for the sake of simplicity, *θ*_e_ and *θ*_o_ were assumed to be equal, denoted as *θ*. However, in practice, to ensure the performance of the filter, certain measures still need to be taken to equalize the two values. In order to equalize *θ*_e_ and *θ*_o_, the primary method involves slotting the inner side of the coupling line. The size of the etched slot for equalization between the two can be obtained via a full-wave simulator. Simulated results of bandstop filter I with different sizes for the etched slot have been plotted in Figure 8. As shown in Figure 8, the size of the etched slot is selected as 4.5 × 1.3 mm^2^ to realize equalization.

**Figure 9:**
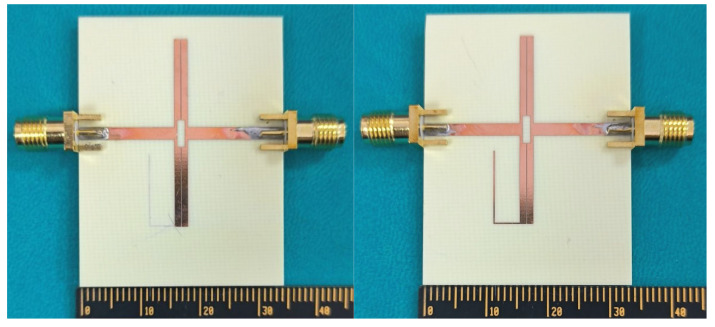
Photographs of broadband bandstop filters I and II.

**Figure 10:**
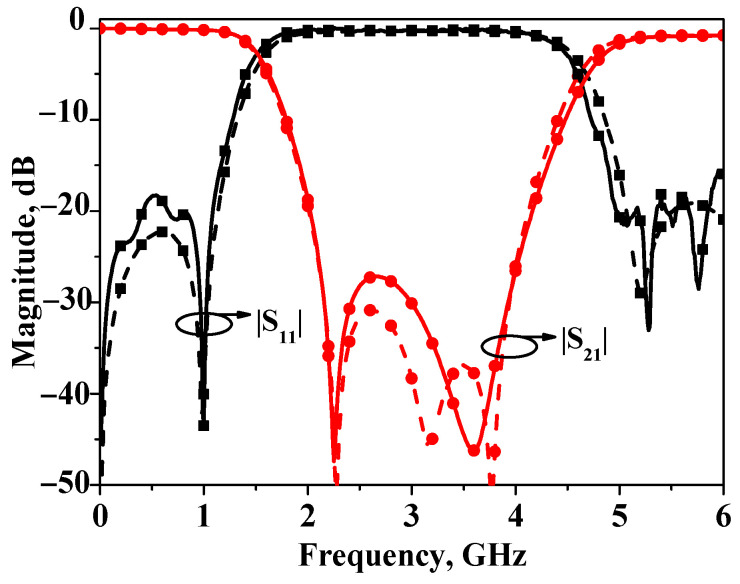
Simulated and measured results of broadband bandstop filter I.

Photographs of the proposed broadband bandstop filters I and II are shown in Figure 9. Based on the aforementioned analysis, the proposed broadband bandstop filter is simulated and designed on an RO4003c substrate with a relative dielectric constant of 3.55 and a thickness of 0.813 mm. The simulated and measured *S*-parameters of bandstop filters I are depicted in Figure 10. The simulation results are obtained by the full-wave simulation software Ansys HFSS 2020 R2, and the testing results are obtained by the Keysight 5071C. As can be seen from the figure, the results match with each other very well. The measured stopband 20 dB insertion loss bandwidth is about 72% (2.02 GHz ~ 4.17 GHz), and the center frequency is 3 GHz. The core circuit size is 0.5 *λ*_g_ × 0.03 *λ*_g_ (31.2 mm × 2.2 mm) (*λ*_g_: the guided wavelength at the center frequency).

**Figure 11:**
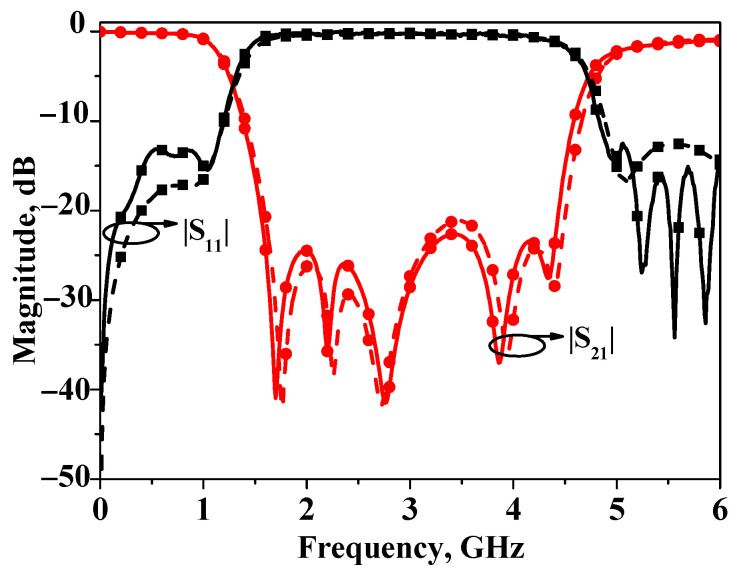
Simulated and measured results of broadband bandstop filter II.

**Figure 12:**
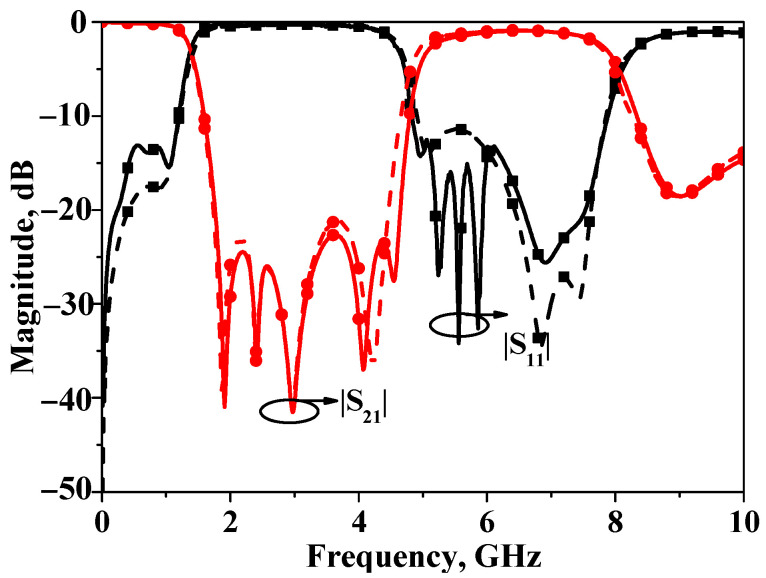
Simulated and measured wideband results of broadband bandstop filter II.

The simulated and measured *S*-parameters of bandstop filters II are plotted in Figure 11. As is illustrated by this figure, the measured stopband 20 dB insertion loss bandwidth is about 100% (1.51~4.52 GHz), and the center frequency is also 3 GHz. The core circuit size is 0.5 *λ*_g_ × 0.1 *λ*_g_ (31.2 mm × 6.5 mm). In order to demonstrate the wideband performance of bandstop filter II, the corresponding response is illustrated in Figure 12.

To further demonstrate the performance of the proposed broadband bandstop filter, comparisons with the previously reported bandstop filters are tabulated and listed in Table 1. As shown in Table 1, among broadband bandstop filters with a stopband bandwidth exceeding 100% and an attenuation level greater than 20 dB, the proposed filter in this paper achieves the smallest size and a relatively simpler circuit structure. This demonstrates the significant advantages of the proposed design in terms of miniaturization and simplicity.

## 4. Conclusions

In this paper, a novel miniaturized broadband bandstop filter with a simple structure is presented. The filter is designed using asymmetrically loaded parallel-coupled microstrip lines, achieving five transmission zeros within the stopband. Closed-form formulas for these transmission zeros are all derived to facilitate practical design. A prototype centered at 3 GHz with a 20 dB rejection bandwidth of approximately 100% is designed, fabricated, and measured, demonstrating the effectiveness of the proposed method. This work provides a comprehensive design approach for compact broadband bandstop filters, highlighting the importance of transmission zeros in enhancing filter performance.

## Figures and Tables

**Table 1:** Performance summary of the proposed broadband bandstop filter and state-of-the-art designs.

REF.	*f*_0_	FBW (Rejection Level)	Transmission Zeros	Layer	Size λ_g_ × λ_g_
2	1.5	138% (20 dB)	3	Dual	0.32 × 0.19
3	2	129% (28 dB)	6	Single	0.5 × 0.45
4	1.5	122% (20 dB)	3	Single	0.37 × 0.25
5	5.5	35% (20 dB)	3	Dual	0.49 × 0.28
14	0.45	47% (40 dB)	3	Single	0.16 × 0.11
15	1.5	100% (20 dB)	3	Single	0.64 × 0.31
16	2	100% (20 dB)	4	Single	0.35 × 0.31
17	2.1	157% (10 dB)	5	Single	1.5 × 0.3
18	1.5	122% (20 dB)	5	Dual	0.31 × 0.2
19	2	50.3% (55 dB)	5	Single	0.3 × 0.25
20	4	50% (20 dB)	3	Single	1.2 × 0.45
21	1.5	67% (20 dB)	5	Single	0.75 × 0.02
22	1	64% (15 dB)	5	Single	0.76 × 0.3
This work	3	100% (20 dB)	5	Single	0.5 × 0.1

λ_g_: The guide wavelength at the center frequency.

## Data Availability

The data presented in this study are available from the corresponding author upon request.
